# Glycemic effects of quinine infusion in healthy volunteers

**DOI:** 10.1186/s13104-017-2744-0

**Published:** 2017-08-24

**Authors:** Audrey Carine Njomatchoua, Aurel Tiakouang Tankeu, Eugene Sobngwi, Jean-Claude Mbanya

**Affiliations:** 1 Cameroon National Obesity Center, Yaoundé Central Hospital, Yaoundé, Cameroon; 2 Faculty of Medicine and Biomedical Sciences, University of Yaoundé 1, Yaoundé, Cameroon

**Keywords:** Quinine, Glucose, Healthy

## Abstract

**Background:**

We aimed to quantify the glycemic effects of quinine in healthy individuals.

**Methods:**

We evaluated the glycemic profile in response to 4 h infusion of 500 ml of 0.9% saline versus 5% glucose solution with and without quinine at therapeutic dose (500 mg) in ten healthy volunteers (8 men) aged 28 ± 9 years. The order of the fourth explorations was randomly assigned. During these explorations, we measured blood glucose every 15 min for 4 h and compared the mean and glycemic fluctuations for each test. A resting ECG was performed before and after quinine infusion in each participant.

**Results:**

The mean glycemic level during the 4-h infusion was 83 ± 5 mg/dl without quinine versus 74 ± 5 ​​mg/dl with quinine (p < 0.001) using saline solute versus 92 ± 7 mg/dl without quinine versus 82 ± 5 mg/dl with quinine (p < 0.001) when associated with the glucose solute. In isotonic dirty solute, quinine induces a cumulative glycemic decrease of 17.5% (p = 0.01) characterized by a nadir estimated at −26.5% at the 60th minute (65 ± 23 mg/dl), p <0.001 followed by a gradual increase until the 4th hour. There were no signs of hypoglycemia or significant prolongation of the QT interval at the ECG. Overall, quinine did not induce a significant change in blood glucose with glucose compared to saline.

**Conclusion:**

The intravenous infusion of quinine at a therapeutic dose induces a light drop in blood glucose with a significant nadir at the 60th minute in the healthy subject without hypoglycemia. This suggests the need for close monitoring in patients at risk of hypoglycemia such as those with severe malaria especially during the first hour of quinine infusion.

## Background

Malaria is one of the most common childhood diseases and a major obstacle for economic and human development in sub-Saharan Africa. It is a leading cause of child mortality and constitutes the main cause of inpatient admission in pediatric wards [1]. According to most recent report, there were 212 million new cases of malaria worldwide in 2015 (range 148–304 million) and the WHO African Region accounted for most global cases of malaria with about 90% of cases [2]. Intravenous quinine although no longer first line of treatment in adults, is one of the mainstay treatments for the severe form of the disease and certainly the most available drug for severe malaria in developing countries [3]. Quinine safety has been established and proven over years. For this reason, it remains the only recommended treatment for severe malaria during the first trimester of pregnancy worldwide due to it safety profile. It is also one of the most widely used molecules, particularly in low- and middle-income countries. However, for decades, there have been a lot of controversy surrounding the potential hypoglycemic effect of this drug [4], considering that, intravenous quinine was always administered using a glucose solution in order to avoid this possible hypoglycemic effect. Recently, the WHO has recommended the use of intravenous quinine in saline solution for the management of severe malaria thus aggravating the already widespread polemic around this topic [5]. However, few studies have investigated the glycemic effect of intravenous quinine in healthy individuals in order to quantify the potential hypoglycemic effect of this molecule. In this light, we aimed to quantify the relative glycemic effects of quinine infusion in order to guide prescription.

## Methods

### Ethical considerations

This study was conducted in accordance with the guidelines of the Helsinki Declaration and was approved by the Institutional Research Ethical Committee of the Faculty of Medicine and Biomedical Sciences of Yaoundé and by the institutional review board of the Yaoundé Central Hospital of Cameroon. All participants provided written informed consent.

### Subjects

Ten healthy volunteers were recruited, 8 males and 2 females, aged between 20 and 32 years with an average body mass index of 23.3 kg/m^2^. The subjects were mostly students with no history of malnutrition and were neither known to be diabetic nor had a family history of diabetes. No subject had clinical malaria at the time of the study. All the subjects had a normal renal creatinine clearance.

### Procedure

Participation was after an overnight fast on four occasions separated by at least 48 h. A standard 4 h protocol was observed on each test day. During the 1st visit, normal saline at 10 mg/kg ideal body weight/min was administered by constant intravenous infusion using a motor-driven pump (i). During the second visit, normal saline was replaced by glucose and the procedure unchanged (ii). On the third visit, normal saline was used with an additional intravenous quinine; quinine dihydrochloride 8 mg base/kg body weight (iii). The 4th visit was similar to the third visit but normal saline was replaced by glucose solution (iv). The order (i), (ii), (iii) and (iv) randomly selected in each case.

On each test day, a cannula was introduced retrogradely into a suitable antecubital vein for repeated venous blood sampling. The cannula was kept patent by flushing with small volumes of sterile heparinised normal saline solution. After 15 min bed rest, two basal samples (at −10 and 0 min) were taken. Immediately after the 0 min sample, the infusion was started through a second cannula inserted in an antecubital vein on the controlateral arm. Further blood samples were drawn every 30 min. Parallelly, capillary blood was collected at −10 and 0 min before the infusion and every 15 min during the infusion on different fingers. This was done in order to control the variability of capillary blood glucose and to ensure that values of this parameter obtained are optimal and reflect real blood glucose values.

At each sampling time-point, blood was drawn for measurement of plasma glucose from venous samples and capillary glucose from capillary samples. In order to verify that all subjects exhibited normal glucose tolerance, the average plasma glucose and insulin concentrations at −10 min and 0 min of the 4 test days was calculated. A 12 lead resting ECG was performed for each participant before and after quinine infusions to estimate the cardiotoxicity of quinine infusion.

All venous samples were collected into dry tubes and sent to the laboratory as soon as they were collected. Following prompt centrifugation, samples were stored at −15 °C before assay.

### Statistical analysis

Data was analyzed using SPSS version 20. Results are expressed as proportions for qualitative variables and as mean ± standard deviation for continuous variables. The Wilcoxon test was used to compare continuous variables obtained with different measures. The area under the curve was calculate for the 4 h of intravenous administration saline and dextrose solution with and without quinine respectively. The Chi square test and the Fisher’s exact test were used to compare categorical variables. Correlations were tested with the non-parametric Spearman test. A *p* value equal or less than 0.05 was considered statistically significant.

## Results

Quinine infusions were all well tolerated. No subject reported having symptomatic hypoglycemia or significant cardiotoxicity during or after any test day of the study.

### Plasma glucose

**Fig. 1:**
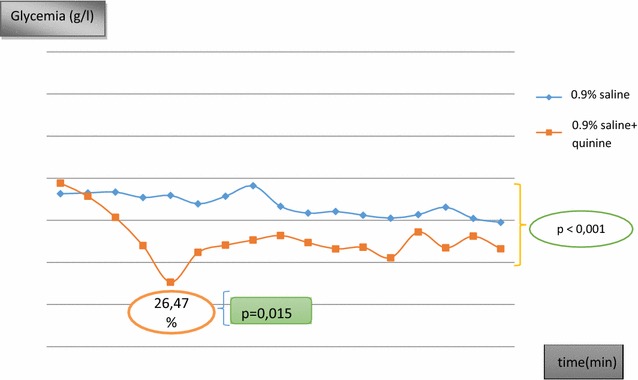
Mean plasma glucose concentrations during a 4 h infusion of 500 ml of saline in healthy volunteers, with a subsequent 4 h infusion of saline with quinine 8 mg/kg body weight

The mean capillary glycemia during 4 h of infusion were 0.83 ± 0.05, 0.92 ± 0.07, 0.74 ± 0.05 and 0.82 ± 0.05 g/l on normal saline, dextrose 5%, normal saline containing quinine and dextrose 5% containing quinine respectively. On the other hand, the matched mean venous glycemia were 0.97 ± 0.02, 0.99 ± 0.35, 0.97 ± 0.06 and 0.93 ± 0.40 g/l, respectively. On comparing the mean capillary glucose, there was a statistically significant difference between mean glucose levels on isotonic saline and those on isotonic dextrose (p < 0.001). The ratio of the area under curve for saline and saline plus quinine infusions was 89%; thus a decrease in blood sugar of 11% induced by quinine over 4 h. At the 60th min, the maximum glycemic drop was observed; 26.5% for quinine in isotonic saline and 7.6% for quinine in isotonic glucose (p < 0.001). There was a significant difference (p < 0.001) between mean values during the 4 h of saline infusion versus saline + quinine infusion as shown in Fig. 1.

Concerning the 12 lead ECG done before and after the administration of quinine in either isotonic saline or isotonic glucose, lengthening of the QT interval was not statistically significant (p > 0.05). None of the healthy volunteers presented symptoms or signs of hypoglycemia during the experimental phase.

## Discussion

This study showed that the use of quinine with saline solution can lead to a slight drop in capillary and venous glucose but which is insufficient to lead to hypoglycemia. The cinchona alkaloids have been prescribed by physicians for hundreds of years but their effect on blood glucose was only reported during the last century [6]. Recent evidence from clinical and laboratory studies has indicated that quinine is a potent stimulant to the release of insulin from the pancreatic beta cells and, in certain circumstances this can lead to hypoglycemia [7–10]. The mechanism by which quinine provokes insulin secretion is known to be through its ability to mimic the action of glucose on potassium permeability of the beta cell membrane, with subsequent calcium influx [11, 12]. However, the beta cell response to quinine could be modulated by the ambient glucose concentrations itself during in vitro studies. In the present study, variations of the blood glucose profile on dextrose and normal saline were not significantly different on venous versus capillary specimens. Previous studies conducted in healthy volunteers and pregnant women found no significant difference between glucose profile under normal saline and saline with quinine (p > 0.05) despite a decrease tendency of blood glucose during quinine infusion [13–15]. Consistent with these observations on a similar population, there was no significant difference when measuring venous blood glycemia, compared with capillary glycemia. Under quinine infusion, there was a critical and significant drop of blood glucose at the 1st hour even though by the 2nd hour blood glucose revealed a tendency to normalize. These findings suggest that quinine is a molecule with a hypoglycemic potential and its effects should be therefore monitored mostly during the 1st hour of the infusion. In addition, intravenous administration of quinine in a patient at risk of developing hypoglycemia must be done with caution since its hypoglycemic tendency can unmask or precipitate hypoglycemia in such patients. Therefore, patients with severe malaria should benefit from venous glycemic measure before using intravenous quinine in saline solution.

Our study present as main limitation the fact that insulin secretion was not assessed. In this context, it is difficult to quantify the whole glycemic effects of intravenous quinine. Also, the small sample size used could have limited the detection of an existing effect.

## Conclusion

Our results suggest that quinine, at therapeutic doses, has a hypoglycemic potential with its maximum effect observed at the 1st hour of infusion but not sufficient to cause hypoglycemia. On the other hand, there is a normalization tendency by the 2nd hour brought about by counter regulatory mechanisms. These observations supported the recent recommendations of WHO on the administration of quinine with saline solution. However, caution must be taken in clinical practice since malaria itself can lead to a decrease in blood glucose and severe malaria can predispose to hypoglycemia. Considering that, administration of quinine must be accompanied by close monitoring in patients at risk of developing hypoglycemia since intravenous quinine can precipitate hypoglycemia in such patients.

## References

[1] Maka DE, Chiabi A, Obadeyi B, Mah E, Nguefack S, Nana P (2016). Economic evaluation of artesunate and three quinine regimens in the treatment of severe malaria in children at the Ebolowa Regional Hospital-Cameroon: a cost analysis. Malar J.

[2] WHO: Malaria. Geneva: WHO. http://www.who.int/malaria/en/. Accessed 6 Jan 2017.

[3] Watsierah CA, Onyango RO, Ombaka JH, Abong’o BO, Ouma C (2012). Provider knowledge of treatment policy and dosing regimen with artemether–lumefantrine and quinine in malaria-endemic areas of western Kenya. Malar J.

[4] Lalloo DG, Shingadia D, Bell DJ, Beeching NJ, Whitty CJM, Chiodini PL (2016). UK malaria treatment guidelines 2016. J Infect.

[5] OMS: Module de formation sur la prise en charge du paludisme. Geneva: WHO. http://www.who.int/malaria/publications/atoz/9789241503976/fr/. Accessed 6 Jan 2017.

[6] Hughes TA. Effects of quinine on the sugar of the blood. Indian J Med Res. 1925;13(2). https://www.cabdirect.org/cabdirect/abstract/19262900483. Accessed 6 Jan 2017.

[7] Harats N, Ackerman Z, Shalit M (1984). Quinine-related hypoglycemia. N Engl J Med.

[8] Looareesuwan S, Phillips RE, White NJ, Kietinun S, Karbwang J, Rackow C (1985). Quinine and severe falciparum malaria in late pregnancy. Lancet Lond Engl.

[9] Okitolonda W, Delacollette C, Malengreau M, Henquin JC (1987). High incidence of hypoglycaemia in African patients treated with intravenous quinine for severe malaria. Br Med J.

[10] White NJ, Warrell DA, Chanthavanich P, Looareesuwan S, Warrell MJ, Krishna S (1983). Severe hypoglycemia and hyperinsulinemia in falciparum malaria. N Engl J Med.

[11] Henquin JC (1982). Quinine and the stimulus-secretion coupling in pancreatic beta-cells: glucose-like effects on potassium permeability and insulin release. Endocrinology.

[12] Henquin JC, Horemans B, Nenquin M, Verniers J, Lambert AE (1975). Quinine-induced modifications of insulin release and glucose metabolism by isolated pancreatic islets. FEBS Lett.

[13] Davis TM, Karbwang J, Looareesuwan S, Turner RC, White NJ (1990). Comparative effects of quinine and quinidine on glucose metabolism in healthy volunteers. Br J Clin Pharmacol.

[14] Dyer JR, Davis TM, Giele C, Annus T, Garcia-Webb P, Robson J (1994). The pharmacokinetics and pharmacodynamics of quinine in the diabetic and non-diabetic elderly. Br J Clin Pharmacol.

[15] Molyneux ME, Taylor TE, Wirima JJ, Harper G (1989). Effect of rate of infusion of quinine on insulin and glucose responses in Malawian children with falciparum malaria. BMJ.

